# Self-referral to inpatient treatment program in a community mental health Centre in Central Norway: investigating the implementation, professionals’ experiences and costs

**DOI:** 10.1186/s12913-021-07273-8

**Published:** 2021-12-06

**Authors:** Inger Elise Opheim Moljord, Kine Gabrielsen Stensvåg, Vidar Halsteinli, Marit By Rise

**Affiliations:** 1 Department of Research, Innovation and Education, St. Olavs hospital, Trondheim University Hospital, Trondheim, Norway; 2 Nidaros Community Mental Health Centre, Clinic of Psychiatry, St. Olavs hospital, Trondheim University Hospital, Trondheim, Norway; 3 Regional Centre for Health Care Improvement, St. Olavs Hospital, Trondheim University Hospital, Trondheim, Norway; 4 Department of Mental Health, Faculty of Medicine and Health Sciences, Norwegian University of Science and Technology, Trondheim, Norway

**Keywords:** Mental health services, Low-threshold, Self-referral, Patient-controlled admission, User participation, Patient participation, Empowerment, Professional’s experiences

## Abstract

**Background:**

Self-referral to inpatient treatment (SRIT) is built on user participation and patient autonomy. SRIT was conducted for patients with severe mental disorders in a Norwegian Community Mental Health Centre. The aims of the present study were to describe the implementation of SRIT, explore the professionals’ experiences of SRIT and assess the costs entailed.

**Methods:**

Qualitative document analysis, interviews with professionals and quantitative analysis of register data from a randomized controlled trial were used.

**Results:**

SRIT seemed to be implemented as intended. According to the professionals, SRIT allowed the patients to cope, be empowered, more active and responsible. Some professionals experienced increased responsibility for patients’ medication and for assessing health and suicide risks. SRIT did not reduce hospital costs. The professionals were satisfied with nurse-led SRIT treatment.

**Conclusions:**

SRIT appears to be a high-quality mental health service that empowers and activates patients. Nurse-led treatment may entail more efficient use of professional resources.

In future implementations of SRIT, the efficient use of service resources and the administration of beds should be investigated. More flexible availability should be considered in line with the intentions behind SRIT, as well as ensuring adequate professional training in assessing health and suicide risk.

## Background

Self-Referral to Inpatient Treatment (SRIT) gives patients the opportunity to refer themselves to short inpatient treatment at their own discretion [1]. SRIT is built on user participation [2], acknowledges patients’ wish to play an active part in treatment and decision-making [3, 4], and is generally recommended by the Norwegian government [2].

During the last decade, SRIT has been implemented in Community Mental Health Centres (CMHC) within specialist mental health services in Norway and abroad for patients with psychiatric diagnoses [1, 5–16]. Here, assessment procedures, length of stay, quarantine between stays, treatment content and follow-up have varied. SRIT may lead to patients experiencing increased predictability, control, autonomy, coping and responsibility [5, 6, 12, 17, 18], normalization of life [17–19], respect by healthcare professionals [11], as well as improvements in life for patients and next of kin [20]. So far, very few studies have investigated the implementation of SRIT in mental health services.

Implementation of new services is highly dependent on the professionals, their motivation and ability to change routines. So far, few studies have investigated professionals’ expectations of and experiences with SRIT [5, 11, 21, 22]. Before establishing SRIT, professionals were sceptical, worrying that patients would misuse the service [5, 22] or that medical assessment was lacking [22]. Experiencing SRIT led to more positive attitudes [11, 12, 21, 22], represented safety for the patients, and the opportunity to self-refer was perceived as advantageous even when not used [11]. More elaborate knowledge of professionals’ perceptions of the potential, benefits and challenges of SRIT is still limited.

Schizophrenia and bipolar disorders are costly due to lengthy stays, relapses and re-hospitalizations [23], worsening the functioning and quality of life for both patients and their families [24]. Effect studies have shown that SRIT does not reduce the number of inpatient days [7, 19] or admissions [7], while non-effect studies show a decrease regarding inpatient days [1, 11, 13, 25].

Self-referral to inpatient treatment (SRIT) entails self-referral; inpatient stays are usually initiated by the patient’s general practitioner (GP) – a procedure that may take some time. Delaying proper treatment may worsen symptoms, potentially resulting in an acute authorization to an emergency unit [26] where a hospital psychiatrist authorizes the admission. Hence, self-referral may reduce pre-admission service costs (e.g. GP involvement). The number and type of inpatient service needed and length of stay may contribute to a reduction of costs. To our knowledge, cost analyses of the SRIT service have not been investigated previously.

The aims of this study were therefore to 1) describe the implementation of SRIT in a CMHC, 2) explore the professionals’ experiences with SRIT, and 3) assess the costs entailed.

## Methods

### Study setting

The aims were investigated through several research methods. The implementation of SRIT (1) was investigated through qualitative documents analysis and interviews with professionals. The interviews with professionals were also used to explore the professionals’ experiences of SRIT (2). The costs of SRIT (3) were assessed through quantitative mapping of service utilization (register data) and calculating the associated service costs.

The study was subsequent to a large randomized controlled trial (RCT) with 53 patients, investigating the effect of SRIT through register data (on inpatient stay, admission) and questionnaires on patient activation, recovery, symptoms, functioning and behaviours [7, 27–31]. The study took place in a CMHC in Central Norway that provides inpatient and outpatient services for a variety of mental health disorders, as well as acute and ambulatory mental health services to 94,000 inhabitants. The CMHC is part of a hospital trust encompassing three CMHCs and somatic health services.

### Data collection, participants and analyses

The different data sources are described in detail in Table 1.

**Table 1:** Data sources

	Implementation	Costs	Professionals’ experiences
Research protocol RCT	X	X	
CMHCs registered number of inpatient days		X	
Inpatient stay costs from the hospital’s information system.		X	
Interviews with 10 professional	X		X

### The implementation of SRIT

Data on the implementation included the RCT research protocol, the CMHC’s written, internal procedures, meeting reports from the supervision group, and qualitative interviews with 10 professionals organizing or providing SRIT were used.

### The professionals’ experiences with SRIT

Data on professionals’ experiences were collected from interviews with 10 professionals, including three focus group interviews and one individual interview. Two focus groups consisted of professionals working as ordinary ward staff taking care of the daily routines and patient care at the ward, conducted five weeks apart at the CMHC in October and December 2016. The third focus group consisted of one key professional with expanded responsibility, the ward leader and a psychiatrist and was added to get a wider perspective of the professionals’ experience with the SRIT service, and was conducted in November 2017. The ward leader had planned to participate in the third focus group interview. However, the leader was prevented from participating during the whole focus group interview. The leader attended the last part and explained that an extraordinary meeting had occurred and caused the delay. The leader continued to express his/her extended experience in an individual interview after the focus group interview.

All professionals that participated in the focus group interviews needed to have at least six months of experience at the ward and be familiar with providing SRIT.

The sample included five specialized mental health nurses (*RPN*), one nurse (*RN*), one specialist social educator, one social worker, one health worker and one psychiatrist. They were between 25 and 65 years old, six women and four men. One of the specialized mental health nurses participated in two focus group interviews since the mental health nurse had extended responsibility in organizing the SRIT offer. To avoid missing important staff on duty, the professionals were placed in groups depending on what day and time suited both the ward and the professionals. The recruitment took place by informing the personnel at the CMHC both orally and in writing, and the ward leader encouraged professionals to participate. Professionals who wished to participate contacted the first and second author. To get sufficient participation, some professional members were directly recruited by asking them in person.

The ward leader agreed to give the professionals time off in compensation if the interview was outside working hours. A total of 21 qualified professionals at the ward were invited to participate; 18 permanent employees and three temporary employees. Seven ward professionals were divided into two focus groups. Later, three professionals participated in the third focus group interview. The small size of the focus group interviews allowed that a few professionals could be absent from the ward at the same time. Each interview lasted between 35 and 45 min.

All interviews were conducted at the CMHC and followed a semi-structured interview guide. The main topics were experiences with the SRIT patients and services, the different roles in the provision of SRIT and perceptions of potential benefits and challenges within the service. All interviews were audio-recorded and transcribed verbatim. Data were analysed according to systematic text condensation, a general cross-case method for thematic analysis of qualitative data [32], involving a general reading, identifying meaning units, merging code groups and reconceptualising into major themes. In the presentation of the results, the overall sample is described as ‘professionals’. Specific professions are emphasized when necessary.

### The costs of health service utilization, including SRIT

Cost calculation was based on service utilization data collected as part of the RCT study [7], where information on outpatient and inpatient services from all CMHC and hospital wards was collected from the hospital patient register system. Unit costs per day and per visit were calculated for all wards from the hospital accounting system, applying a micro-costing approach. Wage-related expenditures accounted for 75 to 85% of the unit costs across wards. Unit costs represent mean costs in 2013 and are reported in USD, applying the exchange rate of 1 USD = 5.875 NOK (monthly exchange rate reported by the Central bank of Norway). The service costs were calculated for each patient by multiplying each inpatient and outpatient episode by the associated ward unit cost. Table 2 shows the list of wards and associated unit costs included. Next, service costs were aggregated into the following categories: SRIT, hospital acute, hospital long-term, CMHC inpatient and CMHC outpatient. Descriptive statistics were used to present cost estimates. Due to skewed cost data, an independent sample T-test based on confidence intervals from non-parametric bootstrapping and bias correction was used to compare cost differences.

**Table 2:** Type of services and unit costs (USD)

Health services	Unit type	Unit cost (USD)
**SRIT**	Per day	656.0
**Acute hospital ward**	Per day	2121.7
**Long-term hospital**		
Ward 3: Psychosis and drug ward-double diagnosis	Per day	1307.1
Ward 4: Complex and non- psychotic disorders	Per day	1647.8
Ward 5: Geriatric psychiatry	Per day	1021.3
Ward 6: Severe mental disorders and malfunction	Per day	1276.6
**CMHC inpatient**		
Ward 1: Short time and crises	Per day	1041.0
Ward 2: Rehabilitation and psychosis	Per day	915.4
Ward 3: Treatment unit for relationship trauma	Per day	1089.0
**CMCH outpatient**	Per visit	423.3

### Ethics

The Regional Committee for Medical and Health Research Ethics in East Norway approved the studies (REK 2009/1704). All participants signed a written consent before taking part in interviews.

## Results

### The implementation of SRIT

The implementation was planned and monitored by the management group, encompassing the head of the division of mental health, the manager at the CMHC, the financial advisor, the RCT project leader, two psychiatrists, the manager of the SRIT ward, the manager of the CMHC’s department for research and professional development, two community representatives and one service user representative. Before the SRIT project started, the leaders of the CMHC and the research project, psychiatrists, and other professionals at the CMHC were engaged in discussions concerning whether SRIT was safe and appropriate. The risk of whether a lack of physician assessment could worsen the patients’ health was debated. Legislations on coercion and patients’ rights [33], shared decision-making [34], ethical principles of doing good, autonomy, and justice [35] were important aspects in the discussions.

Eligible patients were adults diagnosed with schizophrenia or bipolar disorder, some with an additional diagnosis such as reasonably controlled drug addiction. Most patients lived in their own homes, with or without a professional base. All had had previous contact with the rehabilitation unit in continued long-term inpatient or outpatient services for at least two years. The patients either volunteered to SRIT or were recommended by their therapists. An interdisciplinary team assessed exclusions after the criteria: severe substance abuse problems, self-destructive behaviour, inability to consent, or unable to use SRIT as intended. Current inpatients had to be discharged the same day or a few days before they could enter SRIT.

SRIT was implemented in the rehabilitation ward for psychosis, where two rooms were administratively categorized as an independent ward and reserved for SRIT patients.

All SRIT patients received and signed a contract describing how, when and whom they should contact to self-refer. A psychiatrist= approved all patients before they received an SRIT contract. The patients discussed with their therapists what to do when warning signs occurred. Both the patients and professionals were frequently and repeatedly informed about the SRIT procedures, including the possibility of waiting time when the demand was high.

During the RCT, patients could self-refer from Monday to Friday between 8 a.m. and 8 p.m. If they wanted a stay during the weekend, they had to contact the ward before 3:30 p.m. on Friday. All patients were informed about the potential waiting time. Stays could be maximum of 5 days with a minimum of 14 days in between. The 14-day gap between each stay was based on the procedures from the first study in Norway (1) and was implemented to avoid capacity problems and allow patients on the SRIT waiting list to self-refer as soon as possible.

During the RCT, four patients needed longer stays and were re-assigned to ordinary stays. One patient had a long period as an inpatient after drug use. Two patients were transferred to a long-term ward, one to short-term treatment in CMHC and the other to the acute ward. SRIT patients who needed a longer inpatient stay than the acute hospital could offer could not be transferred directly from the hospital but had to use ordinary admission procedures.

After the RCT was closed, SRIT continued, with some adjustments, as part of the ordinary services at the CMHC. Since most patients reported that 5 days was too short to recover, a view supported by the professionals, the maximum length of stay was extended to 7 days. Additionally, admissions during the weekend had to be agreed upon a few hours earlier on Friday, before the afternoon shift, with a 2 p.m. deadline. The patients’ use of SRIT varied from never to very often. The professionals described very frequent use as a potential pathological pattern of regularity and compulsion. Patients who displayed more needs were provided adapted services in cooperation with the therapists.

The specialized mental health nurses were responsible for the consultations with patients at arrival and discharge. They communicated with the patient about their goal for the stay, documented in health records and assessed whether the patient was well enough to use SRIT or needed other treatment. The patients did not have to explain why they contacted the ward since they decided whether the admission was necessary and appropriate, a practice in line with the intentions. Patients were primarily offered milieu therapy entailing participation in the ward’s daily routines and activities. All ward professionals provided support and dialogue if the patient required this. Patients did not receive any specific treatment and were free to organize their stay. The medication plans should normally not be changed, but necessary changes were possible under doctors’ instruction. The patients used the services very differently, displaying different needs. Some patients preferred to shield themselves in their rooms, while others had extensive need for contact and frequent consultations with the ward professionals. Additionally, the SRIT service was used to rest and regulate the daily structure, such as sleep, meals and medication.

### The professionals’ experiences with SRIT

Overall, the professionals were pleased with having SRIT at their ward and believed the service had several potential benefits. SRIT was perceived as a low-threshold service provided in a familiar ward, making it easier for the patients to make contact. Additionally, the professionals described that the service could empower patients who had a strained relationship with the services due to previous experience with being committed. Making their own treatment decisions helped these patients improve their relationship with the services. In addition, the professionals’ view on the capabilities of the patients was strengthened after seeing the patients’ ability to handle the responsibility inherent in the service. This improved the potential for collaboration. Similarly, the professionals perceived that the opportunity to self-refer strengthened the patients’ confidence, helped them to cope at home and enabled them to postpone or even avoid admissions. Sometimes patients cancelled a planned stay, showing that the assurance made the stay unnecessary.

The professionals expressed that SRIT had the potential to prevent acute admissions and longer hospital stays, benefiting both the patients and society in general. Mental health symptoms and social needs occur frequently among some patients with psychosis disorders and drug addiction. A stay could prevent worsening of symptoms and avoid patients being ‘knocked off their perch’ through helping stabilize basic needs with regular meals, sleep and medication, or breaking the isolation in their homes. For some, a stay could prevent mental health symptoms and economic problems due to lack of money and food. According to the professionals, SRIT also reassured the patients’ next of kin since they could help patients realize when they needed a stay, encouraging them to contact the ward knowing they would receive help.

The professionals also described some challenges inherent in the SRIT service. Since a psychiatrist or psychologist did not see the patients at arrival, the mental health nurses were solely responsible for the assessment. If the nurse was unsure whether the patient was well enough to be admitted, the patient could get a second opinion from the psychiatrist or psychologist. Nevertheless, some nurses found the responsibility of SRIT assessment burdening. The health assessment included assessing the suicide risk, described by the nurses as a big responsibility. A manual on how to conduct an arrival consultation, including a suicide assessment scale, was available. This scale was not always used, and the assessment of suicide was conducted differently.

SRIT entailed extra administrational work for the ward professional, especially the mental health nurses. The administration of the patients’ medications was described as labour intensive and challenging. Many patients had help from the community services administering their medications. Although the nurses always asked the patients to bring their medication, medication lists were not always updated in the CMHC’s system. This resulted in extra work for the nurses, who had to ensure that the patients received the correct medicines. Additionally, the ward professionals administered a waiting list, securing the next patient a place as soon as possible. Some professionals found this challenging. In addition, many found the SRIT service incompatible with the current budgetary processes. Ordinary admissions were regarded as an efficient use of resources, while unused beds, to some extent, were regarded to be the opposite from an administrative point of view. Since the service required beds to be available for the patients, offering both ordinary and SRIT admissions was challenging.

### The costs of SRIT

Based on results from the randomized controlled trial, we compared the cost per patient for those who attended SRIT and those who followed the ordinary treatment pathway (treatment as usual, TAU). In Table 3, we report the number of inpatient days, outpatient visits and accompanying costs for both groups. The mean number of days at SRIT was 8.7 at a cost of USD 5727. In total, the mean cost per SRIT patient was 88208 USD (SD 93903) and TAU patient 88186 (SD 91170). There was no difference in mean costs in the 12-month period (USD 21.9; CI^1^: − 50359 – 52507). Looking at the pattern of utilization of different types of services, some indicative differences appeared, although no statistically significant differences were found. Mean days at the acute psychiatric ward were lower for SRIT patients compared to the TAU group. At the same time, mean days at the long-term psychiatric hospital ward were higher for the SRIT group, but the mean number of days was highly influenced by one patient who had inpatient stays amounting to 238 days within 12 months. Excluding this patient from the SRIT group caused the mean number of days at the long-term wards to drop from 14.3 to 5.4 days.

**Table 3:** Health service utilization and costs. Costs in USD. *N* = 53, SRIT = 26 and TAU = 27

		*SRIT N = 26*					*TAU N = 27*				
	*Unit*	*Mean*	*SD*	*Median*	*Min*	*Max*	*Mean*	*SD*	*Median*	*Min*	*Max*
Health Service utilization											
*SRIT*	*Days*	*8.7*	*9.7*	*5*	*0*	*36*	*–*	*–*	*–*	*–*	*–*
*Acute hospital*	*Days*	*3.1*	*6.4*	*0*	*0*	*27*	*7.7*	*18.7*	*0*	*0*	*91*
*Long term hospital*	*Days*	*14.3*	*49.3*	*0*	*0*	*238*	*4.7*	*15.6*	*0*	*0*	*74*
*CMHC inpatient*	*Days*	*47.8*	*70.5*	*24*	*0*	*257*	*50.9*	*68.9*	*19*	*0*	*234*
*CMCH outpatient*	*Visits*	*37.6*	*34.6*	*27*	*2*	*119*	*38.8*	*37.9*	*30*	*0*	*161*
Costs											
*SRIT*	*Per patient*	5727	6351	3280	0	23616	0	0	0	0	0
*Acute hospital*	*Per patient*	6685	13917	0	0	58660	16657	40672	0	0	197706
*Long term hospital*	*Per patient*	15928	52059	0	0	243064	8344	31151	0	0	157006
*CMHC inpatient*	*Per patient*	43960	64433	21512	0	235259	46755	63026	17393	0	214205
*CMCH outpatient*	*Per patient*	15907	14629	11218	847	50375	16431	16050	12700	0	68154
*Sum costs*		88208	93903	52090	10916	336679	88186	91170	60061	0	359063

## Discussion

This study indicated that the patients managed well in using the SRIT service and that the service was safe and valuable to the patients. The fact that mental health nurses assessed the patients at admission had concerned the psychiatrists before the implementation. This contrasted with traditional practice, where a psychiatrist or psychologist consults all patients before admission. Nevertheless, the effectiveness study, which the present study is subsequent to, showed that after 12 months, the SRIT patients did not have worse health outcomes, compared to those receiving treatment as usual [27], a result in line with a previous study [13, 36]. Worries regarding the patients’ health were thus unfounded. We found no difference in cost per patient between the two groups in the 12-month period. On average, the extra SRIT cost was compensated by lower utilization and costs of other services. However, no statistically significant differences were observed between the two groups, except for the SRIT cost itself.

The results showed that the principle of self-referral was upheld when patients did not have to explain why they needed a stay, thus giving the patients easy access to treatment [18], which is in line with previous study [12]. The content of the SRIT inpatient stay, even without any scheduled therapy sessions with a psychiatrist or psychologist, seemed to be adequate. In contrast to usual inpatient treatment, all patients decided what activities they wanted to participate in. This is in line with self-determination, patient’s autonomy and decision-making [3, 4, 6].

The study showed that SRIT beds were perceived as incompatible with the CMHC’s budgetary processes since it could be regarded as a less efficient use of resources. However, the results indicated a potential for reduction in the use of long-term wards. From an administrative point of view, this can outweigh the case of unused beds at the SRIT to some extent. In this project, the implementation did not allow for any fluctuation of demand. The limited number of SRIT beds and potential waiting time may conflict with the principle of SRIT, which is based on autonomy, decision-making and user participation. However, the results showed that the waiting time could sometimes help the patients cope with symptoms, making the admission unnecessary. Nevertheless, more flexible availability of beds should be endeavoured and explored in future implementation of SRIT. In addition, exploring how waiting time affects patients could provide useful knowledge.

This study showed that the professionals believed the SRIT service benefitted the patients. This is in line with previous studies on the patients’ experiences with SRIT [5, 11, 21, 22]. According to the professionals, the opportunity to self-refer was valuable in its own right, even when patients did not use the service. This finding is in line with earlier reports from SRIT implementations, reporting that ‘the bed’ prevented suffering even when it was not in use [25]. Additionally, patients have emphasized the safety inherent in the opportunity [12, 17, 18]. For many persons with severe mental disorders, regular inpatient stays are needed to prevent worsening of symptoms and avoid longer hospital stays. Here, recognition of early signs of deterioration is important [37, 38]. Patients receiving SRIT and treatment as usual had equal health outcomes [27], indicating that the patients are able to assess when they need inpatient treatment to prevent deterioration. This is in line with previous study [13]. This resource should be utilized in future treatment of this patient group. In light of this, the practice of a 14-day quarantine is debatable and might be contrary to the idea behind self-referral, and supports the recommendation to avoid a quarantine period in future projects [13, 39].

The professionals perceived increased empowerment as a potential benefit for the patients. The opportunity to self-refer thus seemed to support the patients in being more active and taking more responsibility during treatment [26]. Previous studies have shown that SRIT patients describe more active cognitive strategies and less resignation, hopelessness and powerlessness [18], as well as increased confidence and ability to cope [17]. SRIT may thus empower and promote self-determination [6]. Additionally, SRIT strengthens the patients’ willingness to ask for help and increases confidence in obtaining mental health care on their own [27]. This is an important aspect of the recovery process [40, 41], suggesting that the SRIT patients become more involved in their own treatment [27]. Leaving the treatment decision to the patients strengthens their participation and autonomy, as well as trains their coping skills [6, 17, 18]. It has been suggested that involvement in treatment is essential to re-establish and preserve the hope of recovery [42–44]. Overall, the SRIT service may be one way to support the patients’ recovery process.

The nurses experienced an increased burden of responsibility for medications since the medication lists not always were updated. This resulted in increased work pressure in obtaining the medicine. Concerns have previously been raised regarding who should have the medical responsibility for SRIT patients during admission [6]. Further use of the SRIT service would benefit from a clearly addressed responsibility for nurses, including assessment of health and suicide risk. According to guidelines for suicide prevention in mental health care, health professionals’ assessment requires competence [45]. Unfortunately, it is impossible to say how frequently an assessment of suicide was conducted without the suicide assessment scale. The author should have investigated this interesting finding during the interviews to get a more accurate picture of it frequency. A potential impact could be that the nurses conducting arrival consultations assessed the risk of suicide in different ways, in the worst case, perhaps not asking for the suicide risk at all, and in that case, enrolling unsuited patients for referral instead of more acute health care.

Further implementation should ensure enough professional training in assessing health and suicide risk as part of an arrival consultation. This is supported by studies showing that taking on new tasks gives new skills but presupposes more and better training, which nurses want to have [36]. That SRIT entailed extra responsibility for the ward professionals, especially the mental health nurses, could be viewed as a transferral of the psychiatrists’ assessment of the patients’ health and need for inpatient treatment. This offset of tasks is supported by a study showing that nurse-led care probably has the potential to provide similar healthcare outcomes as care delivered by doctors [36]. This might lead to reduced costs and better use of professional resources.

Giving patients the opportunity to refer themselves to short inpatient treatment may imply fewer costly hospital stays because of the inherent positive patient outcomes. However, our results did not provide empirical support to this expectation. The service utilization pattern was highly diverse for all service types, from acute inpatient stays to CMHC ward stays. The mean number of days was higher than the median and influenced by highly skewed distributions. The lower mean number of days at acute hospital ward for the SRIT group might be viewed as an indicative promising result, but all in all, we found no support for any cost-saving consequence of implementing SRIT. On the other hand, the fact that no cost increase is observed can give support to the implementation of SRIT – as long as patient outcomes improve.

The present study is one of the first to investigate the implementation, professionals’ perspective and costs of SRIT in the same project. Although the sample of professionals was quite small, it varied in gender, age, professional background and years of experience. Unfortunately, no night shift professional was able to participate. All professionals were recruited directly by the researchers. This constitutes a potential limitation, as it might have made the professional feel ‘forced’ to participate. The mix of three focus group interviews and one individual interview was neither intentional nor optimal. Mixing different interview methods could represent both a strength and a limitation. Here, the main benefit was to add the last key professional to the sample, justifying the pragmatic approach. The first and the third author both worked at the CMHC when the study took place. This might have influenced the participants’ responses and resulted in more favourable evaluations. On the other hand, the professional may have found it more secure to participate in the interviews when they knew the interviewers, compared to an unfamiliar researcher. The data of costs were register data and thus highly valid. The Norwegian health services constituted the context for this study, and the results are thus not necessarily directly transferrable to other contexts or countries with other mental health service systems. Nevertheless, while ensuring the intentions behind SRIT, participation and empowerment, adjustments could be made to adapt to most voluntary mental health service contexts. In this study, the SRIT service was successfully adjusted in line with the experiences from the trial. Since the nurses were given more responsibility for medications and assessment of health and suicide risk, services implementing SRIT require well-trained and experienced nurses.

## Conclusions

SRIT appears to be a high-quality mental health service that helps empower and activate the patients. Several aspects should be discussed in future implementations and studies of SRIT. To attend to the intentions of SRIT, participation and empowerment, the availability of SRIT beds should be increased and quarantines avoided. In a service that entails self-referral and individual assessment of needs in a partnership between professionals and patients, waiting lists seem contradictory. Professionals’ concerns regarding the assessment of health and suicide risk should be met with adequate training to ensure the confidence and safety of both patients and professionals. In future implementation processes of SRIT, such training should be an integrated part. Although treatment costs were not reduced, SRIT is a nurse-led treatment and may thus entail more efficient use of professional resources, which should be investigated more in the future. Future studies investigating the effect of SRIT should also include exploring the patients’ perceptions of potential outcomes and benefits. Their perspectives are key to the good implementation of SRIT in the future.

## Data Availability

Permission might be given from the corresponding author on reasonable request.

## Abbreviations

CMHC: Community mental health centre
RCT: Randomized controlled trial
SRIT: Self-referral to inpatient treatment
TAU: Treatment as usual

## Footnotes

1 Bootstrapping (1000 replicates), bias-corrected confidence interval
